# The Effects of Malnutrition and Refeeding on Perioperative Wound Complications in Surgical Patients With Eating Disorders: A Narrative Review

**DOI:** 10.7759/cureus.110203

**Published:** 2026-06-03

**Authors:** Sheilan Pouri, Harvey N Mayrovitz

**Affiliations:** 1 Medicine, Nova Southeastern University Dr. Kiran C. Patel College of Osteopathic Medicine, Davie, USA; 2 Medicine, Nova Southeastern University Dr. Kiran C. Patel College of Allopathic Medicine, Davie, USA

**Keywords:** anorexia nervosa, bulimia nervosa, eating disorders, malnutrition, perioperative complications, pressure ulcer, protein-energy malnutrition, refeeding syndrome, surgical site infection, wound healing

## Abstract

Anorexia nervosa (AN) and bulimia nervosa (BN) are eating disorders (EDs) with the highest mortality rate of psychiatric illnesses and medical complications, often due to protein-energy malnutrition and micronutrient deficiencies, leading to poor long-term outcomes. Patients with EDs undergo changes in nutritional status that manifest as decreased skin tensile strength, impaired immune function, reduced collagen production, and disrupted electrolyte and fluid balance, thereby increasing the risk of surgical site infections (SSIs), pressure ulcers (PUs), refeeding syndrome (RS), and edema/refeeding edema (RE). This narrative review aims to describe evidence regarding how malnutrition in patients with EDs impairs perioperative wound healing and propose clinical guidelines to reduce complications.

The Web of Science, PubMed, and Scopus databases were searched using terms related to EDs and malnutrition. Peer-reviewed studies were included if they addressed malnutrition in the context of EDs, discussed compromised tissue repair, examined associations between malnutrition and perioperative outcomes, or evaluated nutritional interventions for recovery in malnourished hospital patients. Excluded were opinion pieces and writing that lacked ED wound-healing outcomes.

The main findings of the 31 included studies are as follows. One, chronic malnutrition in AN and BN is associated with dermatological manifestations, such as xerosis and ecchymosis, reflecting reduced dermal strength and systemic dysfunction. Two, laboratory findings, including hypoalbuminemia (<3.5 g/dL), vitamin C deficiency, zinc deficiency, and electrolyte imbalances, are associated with impaired tissue healing and increased risk of infection in this patient population, with hypoalbuminemia a significant indicator of SSI risk. Vitamin C deficiency is associated with impaired collagen cross-linking; supplementation has been linked to accelerating recovery in general wound-healing populations. Zinc deficiency impairs keratinocyte migration and perioperative immunity by reducing CD4+ and CD8+ T-cell production, compounding the risks of RS, SSI, and PU. Three, refeeding malnourished patients carries physiological risks such as RS and RE mediated by electrolyte imbalances and unregulated fluid shifts. Uncontrolled RE is associated with compromised skin turgor, increased tension on sutures, impaired collagen synthesis, and a markedly increased risk of wound dehiscence, PU, and SSI.

The incidence of PU in high-risk surgical patients can be reduced with targeted oral nutritional supplementation. Patients with EDs requiring surgical intervention need specific perioperative care to ensure optimal health outcomes. Preoperative nutrition screenings are essential, and postoperative cautious refeeding plans should include strict electrolyte and fluid intake monitoring. Multidisciplinary coordination, as the standard of care, may help reduce complications such as SSI, PU, and RS and support patients' wound-healing efforts. Future work should reduce the gap in research for this population by testing and developing a standardized nutritional protocol and effective monitoring strategies tailored to eating disorder patients undergoing surgery. Without streamlined care criteria, clinicians must rely on general refeeding guidelines that do not address the unique needs of this patient population, potentially leading to further physiological complications.

## Introduction and background

The lifetime prevalence of eating disorders (EDs), such as anorexia nervosa (AN), is 0.80%, and bulimia nervosa (BN) is 0.28%. They are serious psychiatric disorders in the United States that indicate alarming health concerns for affected individuals [1,2]. Both AN and BN are characterized by a decrease in mental health and impaired physical well-being and are associated with a reduced quality of life that is attributed to malnutrition and higher mortality rates (anorexia hazard ratio: 3.49, 95% CI: 2.43-5.01) [3]. Not limited to psychological dysfunction, AN and BN patients face destructive, life-threatening systemic changes during perioperative surgical intervention [2,4]. Randomized controlled trials (RCTs) examining ED responses to standardized nutritional interventions or surgical risk-prevention plans are extremely limited. Existing evidence is mainly extrapolated from various wound-healing, surgical malnutrition, and pressure ulcer cohorts. Furthermore, a lack of research presents a major barrier to developing science-based strategies for comprehensive treatments.

Nutritional status in ED patients is critical during surgical interventions. It is a key factor in perioperative skin tensile strength (the skin’s ability to resist mechanical stress and tearing), intraoperative complications, and postoperative wound healing. Patient status is affected by inadequate levels of protein (albumin) and micronutrients (vitamin C and zinc), which are vital for collagen synthesis and immune function [5]. In addition, protein-energy malnutrition (PEM) is a condition that is characterized by deficits in both calories and protein that lead to systemic dysfunction. In patients with EDs, PEM can be due to extreme calorie restriction, purging, or both, which can impair collagen synthesis, suppress immunity, and lead to complications such as dermal vulnerability, cardiac arrhythmias, and decreased renal function [6-8]. Though these consequences are well-researched in malnourished surgical patient populations, specifications to AN and BN populations have not been characterized in the literature. Skin becomes brittle, thinner, and more prone to infections postoperatively due to diminished tensile strength [7,9]. Malnutrition is associated with a weakened skin barrier, delayed re-epithelialization, and impaired angiogenesis (formation of new blood vessels), which slows postoperative recovery. Adequate nutrition is essential for angiogenesis, especially postoperatively, when timely skin healing prevents surgical-site infections (SSIs). Consequently, refeeding plans are an essential element of postoperative care, as an influx of adequate nutrition can elicit refeeding syndrome (RS), a dangerous, life-threatening metabolic complication that is triggered by introducing sudden increases in nutrition after long periods of starvation. This rapid reintroduction can cause electrolyte and fluid shifts that lead to life-threatening cardiac, neurological, metabolic, and hematologic complications [8,10].

Rapid refeeding or reintroduction of nutrition after significant starvation (as commonly seen in patients with AN and BN) is characterized by electrolyte shifts, fluid retention, and edema that impair wound-healing mechanisms and can lead to cardiac and neurological failure [6,9,11]. Hypoalbuminemia occurs when serum albumin levels drop below 3.5 g/dL and is commonly observed in ED populations. Hypoalbuminemia is often associated with reduced tissue healing and increased risk of SSIs. This association has been substantiated through spinal surgery patients, where the independent association of hypoalbuminemia preoperatively increased rates of postoperative complications, including longer hospitalizations and surgical wound infections [12,13]. As a result, weakened skin tissue and fluid retention increase vulnerability to pressure ulcers (PUs) and SSIs. This cascade of potential postoperative events underscores the importance of nutritional management and its effects on the entire body and all homeostatic processes. Multifaceted effects after surgery warrant a standardization of confounding variables to score healing outcomes and suggest specific interventions for patients with ED [14-16]. To improve patient outcomes in vulnerable AN and BN populations, integrated care collaboration can address both the psychological and physiological mechanisms at play throughout the surgical process, ensuring positive long-term patient healing rates [4,17].

Therefore, this narrative review aims to deepen understanding of the multifaceted pathophysiological mechanisms that must be considered when patients with ED-related malnutrition and refeeding edema (RE) are being treated. Available evidence predominantly synthesizes general surgical and/or malnourished populations; clinical guidelines on perioperative skin and wound complications in surgical patients with AN or BN are both understudied and limited. As such, this review provides a comprehensive overview of how nutritional disturbances affect both skin and wound healing to properly inform future research, perioperative planning, and targeted protocols of this vulnerable patient population.

## Review

Methods

Search Strategy

This narrative review aimed to synthesize current evidence on how malnutrition in EDs like AN and BN is associated with perioperative wound healing and surgical complications. A narrative approach is preferred over a systematic review due to the diverse methodologies, populations, and outcomes across the studies analyzed. This manuscript did not formally apply the PRISMA 2020 guidelines; however, the selection process is described below to improve reproducibility and transparency. Thus, to deepen understanding of these complex, interrelated topics within this high-risk patient population, a flexible integration of evidence was necessary. Literature searches were conducted for peer-reviewed articles published in English between May 2000 and January 2026 in Web of Science, PubMed, and Scopus databases. The final search was conducted in January 2026 to ensure inclusion of up-to-date data. The primary search string was created using Boolean operators and keyword combinations of “eating disorder AND wound healing AND malnutrition AND refeeding syndrome AND edema." Additional searches were conducted using Boolean operators and a combination of the following keywords: “eating disorder," “anorexia nervosa," “bulimia nervosa," “wound healing," "malnutrition," “refeeding syndrome," "albumin," “surgical site infection," “pressure ulcer," "perioperative," "post-operative," "micronutrient," “collagen production," and "edema." Additional searches combining the listed keywords from relevant studies were conducted to identify additional eligible studies. Reference lists and keywords from foundational articles were also screened to identify additional reference articles related to ED populations in the context of surgery-related outcomes. As this population is frequently understudied in relation to surgical contexts, broader cohort populations were included to help draw conclusions between similar populations and are identified accordingly throughout this piece of literature.

Inclusion and Exclusion Criteria

Inclusion criteria were peer-reviewed original research studies, systematic reviews, case studies, case reports, clinical commentaries, and clinical studies that examined (1) pathophysiological mechanisms linking EDs, specifically anorexia nervosa and/or bulimia nervosa and/or avoidant/restrictive food intake disorder (ARFID), to malnutrition and wound healing complications; or (2) associations between EDs or severe malnutrition and perioperative outcomes such as SSIs, edema, refeeding syndrome (RS), loss of skin integrity; or (3) pathophysiological and cellular-level changes in patients with ED-linked malnutrition, including micronutrient deficiencies, impaired immune function, and angiogenesis; or (4) interdisciplinary nutritional and micronutrient interventions that improve wound healing and post-operative recovery in malnourished AN and BN populations. Clinical commentaries were only used to provide contextual background rather than primary pieces of evidence. Exclusion criteria were editorials, opinion pieces, and studies that did not include EDs or wound healing and its implications/mechanisms.

Results 

A total of 63 articles were retrieved based on initial search criteria. After removing duplicates, titles and abstracts were screened, followed by full-text review of potentially eligible articles. Data extraction was carried out by two reviewers in a standardized manner that analyzed and captured author name(s), study type, patient population, relevant ED and nutrition factors, and associations with RE, SSIs, or PUs. A total of 31 articles were ultimately included in this narrative review that is organized into physiological and clinical sections that address mechanisms underlying skin integrity, delayed wound repair, perioperative care, SSIs, PUs, RS, RE protocols, and perioperative management considerations. Since a narrative design was used, no formal risk-of-bias assessment or meta-analysis was conducted. Instead, findings were organized into thematic sections relevant to the topic: nutritional status, wound healing, perioperative complications, refeeding-related risks in malnutrition, and future implications. Finally, this narrative review was not prospectively registered, and no formal protocol was published before conducting the literature review.

ED-Specific Evidence (Skin Integrity and Wound Healing)

Eating disorders, particularly AN and BN, are often associated with chronic nutritional deficiency that leads to PEM [16]. This malnutrition has the potential to be a deficit in both protein and caloric intake that impairs skin integrity through protein depletion, reduced collagen synthesis, and impaired angiogenesis. This condition slows normal wound-healing processes, reduces collagen's structural integrity, and subjects patients with ED to various health complications. Malnutrition frequently manifests as impaired skin integrity, xerosis, thin skin, edema, and difficulties in wound healing, especially in AN and BN populations [9,11,18]. As the largest organ in the human body, skin integrity alterations in the context of PEM and epidermal atrophy are highly indicative of a systemic immune dysfunction [1,9,18].

In a comprehensive systematic review of publications from 1966 to 2002, Tyler et al. identified 40 distinct cutaneous manifestations in patients with AN and BN, emphasizing the crucial role that dermatological signs play in the early recognition of a deeper-rooted systemic issue [1,19]. A cross-sectional study involving 200 ED patients found xerosis, ecchymosis, and lanugo hair, indicating the prevalent effects of chronic malnutrition on the skin [5]. Specifically, 96.7% of AN patients (95% CI: 88-99%) and 84.42% of BN patients (95% CI: 75-88%) exhibited symptoms of xerosis (presenting as dry, rough skin), indicating epidermal barrier disruption and weakness of the natural moisturizing factor (NMF) [20]. The NMF is composed of several components, including amino acids, inorganic salts, and pyrrolidine-3-carboxylic acid. When NMF integrity is compromised, skin barrier function deteriorates, resulting in loss of epidermal moisture, which is associated with the exacerbation of perioperative wound-healing time and infection risk [5,20]. The rates of ecchymosis (85%; 95% CI: 74-92%) and lanugo hair (77.9%; 95% CI: 68-85%) in AN patients further reflect vascular fragility and protein deficiency [1,5].

Preoperatively, ecchymosis can cause coagulation dysfunction, often stemming from PEM-related platelet impairments that produce unstable fibrin clots and compromise hemostasis of surgical sites. This condition increases the risks of intraoperative bleeds and susceptibility to surgical wound contamination postoperatively, attributable to the compromised integrity of keratin, collagen, elastin, and connective tissue and disrupted skin repair mechanisms [1,5,20]. These manifestations collectively serve as visible indicators of nutritional deficiencies present in the ED population that drive immune dysregulation and disrupt wound healing.

​​​Macronutrient and Micronutrient Deficiencies in Wound Healing (Evidence Extrapolated From Surgical Malnutrition Literature)

In addition to dermatologic indicators, ED patients commonly exhibit dysregulated physiological mechanisms attributed to macronutrient and micronutrient deficiencies. Amino acids are the building blocks of protein essential for collagen synthesis and are depleted in ED patients [20]. This is due to inadequate protein intake, which results in insufficient amino acid building blocks essential for maintaining healthy collagen and skin elasticity. Thus, PEM is indicative of essential nutrient limitations that impair skin healing at the surgical site, hindering proper tissue remodeling [18]. Both protein and caloric intake are vital for the energy-intensive process of wound healing, with energy requirements averaging 30 to 35 kcal/kg across all stages of skin repair. This number increases to approximately 40 kcal/kg for malnourished patients, including those with AN and BN. Protein requirements are 1.2-2.0 g/kg/day [11,21]. Already prone to perioperative infections and longer hospital stays, PEM populations face magnified challenges consequent to the greater protein intake requirements necessary for optimal wound healing mechanisms [9]. In parallel, 1 ml/kcal/day is the recommended fluid intake for patients with wounds and should be adjusted according to existing health conditions, such as AN or BN. Optimization of nutritional status is recognized as a key factor for reducing postoperative complications. Moreover, fluid intake is crucial for maintaining low blood viscosity and adequate skin elasticity. Preserved skin turgor helps protect against the potential development of xerosis, skin breakage, and, ultimately, pressure-related ulcers [11,21].

Another key constituent of collagen synthesis, as it relates to skin integrity, is albumin, the most abundant protein in blood [20]. Its deficiency may lead to reduced skin tensile strength, and hypoalbuminemia is a strong predictor of malnutrition and poor surgical outcomes [22]. While hypoalbuminemia is associated with poor surgical outcomes, it is also influenced by factors such as inflammation and fluid status. It should not be used in isolation as a marker of malnutrition. A prospective study including 130 surgical patients found that 83 with hypoalbuminemia (<3.5 g/dL) developed considerably more perioperative complications (39.8%) compared to 47 patients with normal albumin labs (8.5%; p=0.000318) [23]. Of the 83 hypoalbuminemia patients, 24 (28.9%) had tissue edema-induced surgical site infections, with nine cases of dehiscence due to impaired tissue repair pathways. In contrast, of 47 patients with normal albumin levels, only four (8.5%) experienced these complications (p=0.000318) [23]. In a 2024 systematic review and meta-analysis, 43,059 hip fracture patients with hypoalbuminemia (45.3% with <3.5 g/dL) were observed to have a statistically significant increased risk of any surgical site infections (OR 1.25, p=0.008), with a greater association between deep surgical site infections (OR 1.76, p=0.05) rather than superficial surgical site infections (OR 1.06, p=0.77) [22]. Two additional meta-analyses in populations undergoing spinal surgery obtained similar results, confirming the significant connection between preoperative hypoalbuminemia and increased likelihood of postoperative complications (wound infections; prolonged hospital stays) and further reinforcing the cross-surgical relevance of ensuring optimal nutrition status [12,13]. While these analyses exclusively examined orthopedic hip fracture patients, the pathophysiological findings of hypoalbuminemia can be cautiously applied to the broader scope of surgical patients with increased skin infection risk, including ED patients undergoing surgical procedures.

Parallel to albumin’s role in wound healing, micronutrients like vitamin C and zinc are of equal importance in collagen synthesis and in supporting immune defense, offering protection against AN and BN clinical malnutrition markers, including xerosis, ecchymosis, and surgical site infections. Vitamin C is both a water-soluble vitamin that acts as a cofactor for hydroxylation reactions critical to cross-linking collagen fibers and a fundamental player in immune defense. Deficiency can manifest as easy bruising and bleeding, scurvy, and delayed healing, which precipitates perioperative complications such as brittle, thin skin leading to surgical wound complications and longer hospital stays [11]. A 2023 systematic review of a general wound-healing population observed vitamin C’s role in wound rehabilitation and found that supplementation with vitamin C resulted in a 3.94 times increased likelihood of achieving an accelerated recovery than that of a placebo (OR 3.99, 95% CI: 2.06 to 7.73) [24]. These findings have not yet been validated in AN or BN populations and should be applied cautiously when extrapolated to these groups. Similarly, zinc helps protect malnourished surgical patients against inflammation. It drives cell proliferation and tissue repair via keratinocyte migration and accelerates epithelial cell regeneration [7,11,18,20]. Sufficient levels of zinc also support perioperative immunity by increasing CD4+ and CD8+ T cell generation and by enhancing albumin binding, since albumin accounts for 10% of plasma proteins [20]. Through their interplay and cumulative effects, albumin, vitamin C, and zinc collectively create bridges among nutrition, skin healing, and immune strength (Table 1). These findings suggest that a deficiency in a single component can lead to direct and indirect physiological imbalances, including immune dysfunction, malnutrition, delayed healing of surgical incisions, and various perioperative infections [6,9].

**Table 1:** Key nutrient deficiencies, their roles in wound healing, and associated perioperative risks in surgical patients with EDs This table (created by the authors using Microsoft PowerPoint (Microsoft Corp., Redmond, WA, USA)) summarizes key nutritional deficiencies commonly seen in surgical patients with EDs and how these deficiencies cause dysfunctional wound-healing mechanisms and increase surgical risks. EDs: Eating disorders, RS: Refeeding syndrome, RE: Refeeding edema, SSIs: Surgical site infections, PEM: Protein-energy malnutrition, PUs: Pressure ulcers

Nutritional deficiency or biomarker	Function in wound healing mechanisms	Associated complications in surgical patients with eating disorders
Hypoalbuminemia (<3.5 g/dL) [12,13,22,23]	Oxygen delivery to, amino acid transport, immune support	SSIs, wound dehiscence
Vitamin C deficiency (<0.2 mg/dL) [11,24]	Collagen cross-linking, collagen synthesis, immune defense	Scurvy, scurvy-like bruising, delayed wound healing, PUs
Zinc deficiency (<70 µg/dL) [7,11,20]	T-cell production, immune defense, keratinocyte migration, epithelial regeneration	Inflammation, poor immune function, infection
Electrolyte imbalances (low phosphate, potassium, magnesium, sodium, and fluids) [6,8,25]	Fluid balance, cell membrane stability, adenosine triphosphate production	RS, RE, hypoxia, SSI, cardiac failure
PEM [6,9,11,16]	Collagen synthesis, fibroblast proliferation, angiogenesis, immune function	Xerosis, RE, PUs

SSIs Due to Malnourishment and Immune Dysfunction (Evidence Extrapolated From Surgical Malnutrition Literature)

Nutritionally depleted ED patients exhibit evidence of increased susceptibility to postoperative SSIs. Weakened skin tensile strength is associated with wound dehiscence, opening a route of infection at the SSI. Preoperative ecchymosis further contributes, as it is directly linked to hematoma formation, delayed incision wound healing, and increased risk of SSIs [9,17,23]. Moreover, patients with AN and BN exhibit impaired immune response with decreased T-cell activity and antibody, which may leave them vulnerable to postoperative SSIs and hospital-acquired pathogens [11]. Partial prevention of these infections relies on assessing preoperative nutrition by utilizing Subject Global Assessment tools and frequent nutrition tests throughout the patients’ perioperative course [25]. In addition to these skin infections, patients with EDs are at increased risk of PUs due to weakened skin barriers and PEM, which contributes to decreased collagen synthesis.

PUs Due to Compromised Skin Barrier Function (Evidence Extrapolated From Surgical Malnutrition Literature)

Nutritionally depleted ED patients also exhibit evidence of increased susceptibility to the development of PUs. These ulcers are defined as localized superficial and deep tissue injuries, characterized by easy skin tears, which have been documented in 96.7% of AN patients, and ecchymosis, which has been observed in 85% of AN patients. This form of postoperative injury typically arises from friction and sustained pressure and is exacerbated by malnourishment [9,16]. Pressure ulcer stages are observed to advance as protein levels decrease in the body: stage I ulcers indicate minimal tissue loss, while stages III and IV are consistent with severe disruption of cutaneous tissue, typically characterized by moderate to severe levels of nutritional deficiency [9]. A 2020 prospective hospital-based study in South Korea reported a PU prevalence of approximately 14.8% among 160 patients with PEM. Extreme weight loss was consistently found as an independent predictor of global postoperative surgical complications and prolonged hospital stays, including the development of PUs [9,15,16]. Thus, AN and BN patients are predisposed to skin breakdown and infection during postoperative immobility [5,20]. In a subset of a large systematic review, 13 RCTs comprised of 1,782 malnourished adults (ages 35 to 87 years; mean age 67) with varying degrees of nutrition deficiency demonstrated that oral nutritional supplementation significantly reduces the rate of postoperative complications compared with control groups (OR 0.73, 95% CI 0.57-0.94) [26].

In the full review of 44 RCTs (n=5,716) with elderly malnourished adults, oral nutritional administration of approximately 588 kcal/d (22 g protein/day) for an average of 74 days was associated with improved postsurgical outcomes (OR 0.68, 95% CI 0.59-0.79) [26]. Outcomes included fewer cases of PUs and SSIs and strengthened wound healing [26]. It should be noted that out of these 44 RCTs, the populations did not include patients with EDs; therefore, they should serve as extrapolated data that can be applied to malnourished ED surgical patients in the absence of clinical evidence for this understudied population. In parallel, a meta-analysis observed that the introduction of oral nutritional supplementation (250 to 500 kcal per serving) delivered over two to 26 weeks reduced the incidence of PU development in at-risk, malnourished elderly patients by approximately 25% [9]. Reflecting these findings, the National Pressure Ulcer Advisory Panel recommends that severely malnourished patients with stage III or IV PUs receive extensive monitoring and increase caloric intake by 35 to 40 kcal/kg/day to support anabolism and tissue growth and to reduce the likelihood of RE [9].

RS Overview and Pathophysiology 

When severely malnourished ED patients receive rapid, dense nutritional supplementation over a short period of time, they are at high risk of RS. This risk emerges as their metabolism abruptly shifts from a sustained catabolic state to a carbohydrate-induced, insulin-driven anabolic state, leading to significant electrolyte shifts and fluid retention. Glucose, phosphate, potassium, thiamine, and magnesium are funneled into cells to drive cellular processes, including ATP production, glycogenesis, and collagen synthesis, depleting serum levels [25]. Concurrently, carbohydrate-induced renal sodium and water retention expands extracellular fluid volume, leading to fluid overload and peripheral refeeding edema in ED patients. This sudden metabolic transition in the body often gives rise to major electrolyte imbalances in vulnerable ED patients, typically seen within 72 hours of nutrition reinitiation. The Australasian Society of Parenteral and Enteral Nutrition (AuSPEN) has a similar timeline that emphasizes the necessity for RS monitoring to begin immediately upon the start of caloric intake and extends through the first several days of postoperative status [6,25,27].

According to one study, RS was observed to affect approximately 6% to 10% of hospitalized patients suffering from malnutrition [6]. In a dedicated AN hospital unit (median BMI of 13.1 kg/m²), it was reported that 45% of patients developed hypophosphatemia and other electrolyte imbalances [28]. This finding emphasizes that monitored, gradual caloric intake is central to preventing refeeding syndrome and leading to shorter hospital stays [10]. These findings underscore the critical role vigilant monitoring of nutrition status plays in refeeding undernourished patients.

Additionally, the varying risk profiles of ED patients contribute to the severity of RS, underscoring the need for tailored clinical management as an essential component of perioperative care. This is seen in children and young adolescent patients, where the management of restrictive eating disorders requires specific considerations such as caloric adjustments based on developmental status and age [29]. One narrative review provided evidence that severe AN patients who had inpatient protocols with structured caloric intake targets and surveilled daily electrolyte intake had improved weight restoration safely and effectively [30]. Overall, refeeding is the critical first step to postoperative recovery of undernourished ED patients; however, optimal outcomes rely heavily on tightly regulated refeeding schedules and gradual weight gain [6,10,25,28]. Both factors can potentially be crucial for predicting long-term nutritional recovery outcomes at one year. Electrolyte monitoring should be performed every 24 to 28 hours during the first week following an operative procedure, as the risk of refeeding syndrome is greatest at this stage. Nonetheless, gaps in electrolyte supplementation guidelines result in no standardized clinical practices [6,10].

Electrolyte Dysregulation in RS

The cardinal sign of refeeding syndrome is hypophosphatemia, which causes a catastrophic domino effect of physiological dysregulation during refeeding after surgical procedures in ED patients. Phosphate levels drop because of excessive vomiting, exercise, laxative/diuretic use, or binge eating [6,25,28]. In a brief editorial commentary, it was suggested that without tight regulation of phosphate supplementation, the prevalence of RS and RE with restrictive eating disorders has the potential to increase and contribute to severe malnutrition complications [31]. It should be noted that the conclusions from this editorial should be utilized as preliminary and thought-provoking rather than hard evidence. That said, in recent years, clinical protocols have implemented controlled refeeding strategies to mitigate RS and edema risks [8,10]. 

A multicenter randomized trial in 13 ICUs across Australia and New Zealand found that a temporary restriction in calories (feeding critical-condition RS patients 20 kcal/h for two days) improved three-month survival outcomes compared to standard feeding models [8]. The National Institute for Health and Care Excellence (NICE) in the United Kingdom developed evidence-based clinical guidelines to improve the quality of patient care. Regarding refeeding, NICE recommends starting nutrient intake at no more than 50% of the individual's caloric energy requirements. Malnourished patients (BMI ≤14) are recommended to begin with the lowest caloric intake and gradually increase over four to seven days while monitoring electrolyte levels and cardiac function [6]. Similarly, the 2025 AuSPEN consensus statement on refeeding syndrome urges individualized caloric intake rates, routine electrolyte protocols, and multidisciplinary engagement to be core components in preventing RS in high-risk patient populations, such as those with EDs [27]. In a retrospective study consisting of 633 severely malnourished AN and ARFID patients (mean BMI 14.1 ± 2.1 kg/m²), 39% (247 patients) of the cohort who received phosphate supplementation were observed to have greater weight gain, further signifying the importance of careful nutrition regulation postoperatively [31].

**Figure 1:**
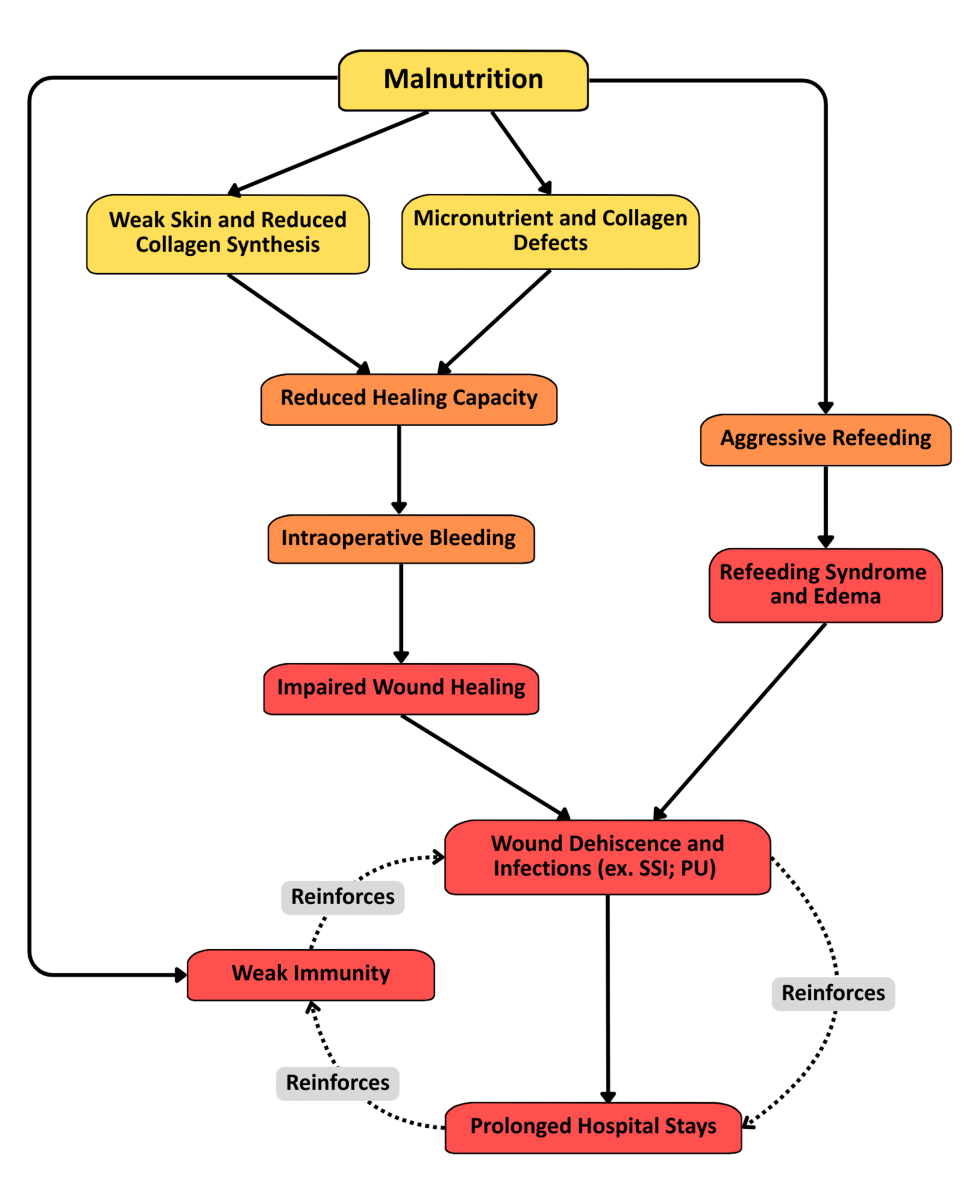
Pathophysiological cascade linking ED-associated malnutrition to perioperative complications and infection loops The yellow pathway depicts preoperative chronic malnutrition. These deficiencies manifest as intraoperative consequences, indicated by the orange pathway. Red pathways depict postoperative effects on immunity, in addition to prolonged inflammation and fluid retention. Dashed arrows are indicative of reinforcing loops between prolonged hospital stays, further weakening immunity, and perpetuating infections. ED: Eating disorder, SSI: Surgical site infection, PU: Pressure ulcer This illustration was created by the authors using Canva (Canva Pvt. Ltd., Sydney, AUS) sans AI generative tools.

Specifically for postoperative ED patients, SSIs and PU risk are amplified due to compromised skin integrity combined with impaired wound fusion. This leads to elevated soft-tissue swelling due to increased soft-tissue blood perfusion and is characteristic of RE. Together, these postoperative complications reinforce a pathophysiological cascade of bleeding, wound dehiscence, prolonged hospital stays, and weakened immunity. Figure 1 illustrates the overall pathophysiological cascade in which preoperative ED-linked malnutrition causes intraoperative and postoperative complications. The preoperative pathway (yellow boxes) depicts how chronic malnutrition causes a reduction in skin integrity, collagen synthesis, hypoalbuminemia, and micronutrient defects (vitamin C and zinc) that decrease overall healing capacity. The intraoperative pathway (orange boxes) depicts intraoperative bleeds due to poor clotting, reduced platelet function, and limited vessel wall collagen. The red boxes depict the direct postoperative effects of nutrition on immunity, as well as the consequences of aggressive refeeding protocols. Impaired wound healing in combination with RS triggers wound dehiscence, infections (SSI/PU), and longer hospital stays. Dashed arrows are indicative of reinforcing loops between prolonged hospital stays, further weakening immunity, and perpetuating infections. These pathways form a vicious cycle of chronic malnutrition, recurrent infections, and increasing mortality rates in this vulnerable patient population. Effective management of RS thus requires vigilant monitoring of electrolyte levels and tailored refeeding protocols to minimize the risk of complications and improve long-term patient outcomes [11,31].

Perioperative Management (Impact of Edema on Wound Healing)

As tissue surrounding a surgical incision site begins to swell, the risk of stitches being pulled or wounds reopening increases. After a surgical procedure, overfeeding ED patients can cause detrimental electrolyte and fluid shifts that add approximately 1 kg of plasma expansion over approximately four days. These shifts can compress blood vessels and may be misinterpreted as improved healing. Also, the associated reduced oxygen delivery impairs collagen synthesis and epithelialization in tissues, which favors a cascade of impaired immune function, facilitating bacterial proliferation and increasing SSIs. [9,19]. Edema, therefore, can establish a pathophysiological loop of inflammation, stagnant fluid causing edema, soft-tissue breakdown, infection, and prolonged malnourished hospital stays [6,19].

In addition to RS, close caloric monitoring, starting small at 10 kcal/kg/day within the first 24 to 72 hours, is recommended to prevent edema and improve long-term outcomes. Daily wound assessments, frequent bloodwork, tracking patient intake/output, and consistent labs, including renal function and insulin resistance tests, are vital to personalize refeeding protocols and prevent delayed wound healing and life-threatening complications postoperatively. These highly integrated electrolyte shifts underscore the vitality of individualized refeeding plans to minimize the likelihood of developing RS and improving long-term metabolic outcomes [25,26,31].

Discussion

Nutritional Rehabilitation and Preventing Complications

The review’s findings emphasize the fragility of interactions between malnutrition and skin integrity in patients with EDs such as AN and BN. Complications include increased risks of SSIs, PUs, and dehiscence, all of which are associated with malnutrition, which is linked to a weakened skin barrier and impairs collagen synthesis and the rate of angiogenesis [7,11,14]. Moreover, undernourished ED patients have dermatological manifestations such as xerosis, ecchymosis, and lanugo, which, when combined with hypoalbuminemia and micronutrient deficiencies, can be detected in hospital labs and serve as clinical markers of malnutrition severity [16,20,21].

The metabolism of malnourished ED patients shifts from a catabolic to an anabolic state, causing electrolyte shifts and leading to RS. Refeeding syndrome extends beyond a phosphate deficiency, encompassing abnormal sodium and fluid balance, alterations in fat metabolism, and deficiencies in magnesium, potassium, and thiamine [6]. Increasing carbohydrate intake triggers a surge of insulin, which drives renal sodium and water retention by suppressing atrial natriuretic peptide (ANP) and B-type natriuretic peptide (BNP), thereby decreasing urine output. This complex consequence explains how RE remains common in malnourished patients when strict nutrition protocols, such as those established by NICE and AuSPEN, are not followed [6,27,31]. Thus, RS is characterized by a plethora of risks ranging from fluid imbalances to cardiac and renal risks to compromised skin integrity. The likelihood of ED patients developing infections and ulcers is also heightened, further emphasizing the need to develop and adhere to protocols such as the NICE guidelines.

Aggressive refeeding postoperatively, however, can lead to severe complications, including dangerous electrolyte shifts that promote refeeding syndrome, as well as increased suture tension and reduced oxygen delivery to tissues. This sequence leads to edema and creates a direct route for bacterial pathogens to enter the incision site [17,21,32]. Findings of this narrative review advocate for standardized guidelines and SGA screening tools to reduce the prevalence of these complications in ED patients [16,33]. Frequent lab monitoring and individualized refeeding plans restore necessary nutrients to the body in a controlled manner to minimize postoperative wound complications. Future research should evaluate various refeeding models, such as oral and enteral, to determine which is best tolerated in surgical populations with AN and BN [17,26]. Moreover, comparative studies examining high versus low caloric refeeding approaches should be conducted, as this evidence is currently lacking and is only present in non-surgical cohorts that suggest higher caloric intake could reduce both RS and length of hospitalization [34,35].

*Perioperative Management for *Clinical Implications

Refeeding syndrome manifestations are typically highest within the first 72 hours of nutrition reinitiation; therefore, electrolytes should be monitored at least once daily during the first week of refeeding, with more frequent monitoring (e.g., every six to 12 hours) in high-risk AN and BN patients [6,25]. This course of action is vital to prevent life-threatening complications and optimize wound healing mechanisms for undernourished ED patients [9,26]. Clinically, this review calls for an increase in the number of scoring tools used, such as the Malnutrition Universal Screening Tool (MUST) or the SGA. Guidelines are needed to optimize preoperative nutritional supplementation so that the health of a patient with ED does not spiral into severe postoperative complications [11,33].

According to the United Kingdom’s NICE guidelines, strict monitoring of electrolytes combined with incremental caloric increase can help nutritionally restricted individuals achieve expedited weight gain (0.43 to 0.86 kg/week) and achieve full remission within 12 months [6,26]. Supporting these recommendations, the 2025 AuSPEN consensus statement emphasizes essential components of safe refeeding include individualized phosphate replacement, electrolyte replacement, and multidisciplinary team involvement to reduce rates of postoperative complications for populations with comorbidities [27]. Thus, to prevent postoperative complications such as RS or PUs, it is imperative for at-risk patients to receive suitably frequent surveillance and comprehensive care. Several analyses emphasize that the key to improving perioperative patient outcomes lies in multidisciplinary coordination among dietitians, psychiatrists, surgeons, and endocrinologists. This is specifically important for pediatric and young adolescent patient populations with restrictive EDs such as ARFID and AN, where supporting patient growth and age must be considered in refeeding protocols to optimize nutritional intake while reducing RS and RE risks [29,30]. Close collaborations ensure personalized care protocols consisting of sustained monitoring and nutritional supplementation [9,15,20,25].

Summary of Key Clinical Recommendations

Preoperative: Nutritional screenings via the use of validated assessments and tools such as the SGA or MUST are essential to assess potential nutritional deficits in surgical ED patients [11,25,33].

Perioperative: Multidisciplinary care through coordination between psychiatrists, surgeons, dietitians, psychologists, and endocrinologists is necessary to ensure that patients with ED are closely monitored and receive individualized care [9,15,20,25,29,30]. Micronutrient supplementation of vitamin C and zinc should be replenished preoperatively and postoperatively to support immune function and collagen synthesis [7,11,20,24]. Serum albumin levels should be tracked preoperatively as a risk predictor of impaired wound healing. Postoperatively, it serves as a potential influencer and indicator of nutritional status; unregulated, it can lead to complications such as PU, SSI, and RE [12,13,22,23].

Postoperative: Given that RS often manifests within the first 72 hours of caloric reinitiation, electrolytes should be monitored every six to 12 hours for the first week postoperatively in high-risk AN and BN patients. Fluid intake and output, along with renal function, should also be surveilled [6,25,27]. Refeeding protocol per the NICE and AuSPEN guidelines should be established. Initiating refeeding at 10 to 20 kcal/kg/day per the NICE guidelines, with gradual increases targeting weight gain of 0.43-0.86 kg/week. The AuSPEN guidelines support this framework and add that electrolyte replenishing should be individualized [6,10,27]. Daily wound assessments should be conducted daily, concurrent with electrolyte monitoring, to rule out potential infection risks that increase the length of hospitalization [9,11,25,26].

Strengths of This Review

The strengths of this review include a cohesive synthesis of three understudied domains of health: the clinical vulnerability of patients with eating disorders in an operative context, the intricate interplay between perioperative wound-healing mechanisms and nutrition, and the risks of RS, including edema. Diverse evidence highlights the paradoxical impacts of rapid weight gain on the intricate physiology of wound healing, displaying how this process can incidentally lead to perioperative complications.

The crucial relationship between nutritional status and surgical outcomes was also thoroughly studied in this review, specifically, how biological processes involved in wound healing rely on adequate nutrition to promote long-term outcomes. Given the extensive range of nutritional profiles ED patients present with, evidence has underscored the importance of assessing comprehensive nutrient profiles early to enable tailored patient care. Notably, studies that included integrated patient care models illustrate how collaboration fosters enhanced communication among providers by drawing on the expertise of each specialty. In addressing the complex needs of patients with EDs, care teams can reduce the incidence of perioperative complications.

Overall, the dermatological, surgical, and nutritional findings support a systemic shift in perioperative care to improve outcomes for patients with EDs by interweaving team-based models and interdisciplinary communication. This review highlights the urgent need for further research into the effective monitoring and management of malnourished patient populations in surgical settings to improve patient outcomes.

Limitations

This review has several limitations that should be considered. First, there are few RCTs recommending standardized refeeding programs for malnourished and restrictive ED patients, as well as a lack of criteria for electrolyte correction or reintroduction. This inconsistency has led to a wide range of clinical studies consistently finding that tight regulation and controlled supplementation yield the best patient outcomes [26]. Moreover, as this is a narrative review, no formal risk-of-bias assessment was performed. The design of this review was to synthesize thematic patterns in literature that can potentially be applied to an understudied patient population, rather than to provide a hierarchy of evidence.

Additionally, literature on perioperative wound protocols and studies relating skin integrity to wound-specific nutrition for patients with BN and ARFID was both scarce and broad in treatment avenues. Most ED research data were derived from small AN cohort studies, limiting generalizability across ED subtypes, despite their similarities. Furthermore, a sizable portion of the cited evidence was collected from general malnutrition in a surgical setting, elderly patient cohorts displaying malnutrition and PUs, orthopedic surgical patients, and general wound-healing cohorts.

Though the findings of this review are both pathophysiologically related, clinically relevant, and clearly identified, the absence of direct perioperative data limits the strength of clinical recommendations. Moreover, this review included a small number of clinical comments used as background information and potential future directions, which are subject to author bias. Future cross-comparative studies are necessary to develop strategic perioperative protocol guidelines tailored to contrasting ED symptoms. Finally, as a narrative review, this writing can only provide general background information and draw conclusions from previously completed studies, rather than offering a meta-analysis or quantitative conclusions on its own. Literature selection is susceptible to bias depending on the author’s interpretation, potentially limiting the replicability of this review.

Research Gaps and Future Directions

While this paper’s findings underscore the connection between perioperative ED wound healing and nutrition management, future studies should address the research gap to optimize outcomes for this subset of malnourished patients. A retrospective cohort study found the efficacy of wound-specific oral nutritional supplementation to be 61% versus 34.5% in the control group, noting the greatest wound healing benefits were in the first and second weeks of the healing process [33]. Research should include RCTs with restrictive and bingeing ED patients that examine various nutritional protocols for surgical wounds. In doing so, protocols can be standardized for ED patients undergoing surgical procedures.

Another area warranting exploration is tailoring macronutrient ratios, including vitamin C, zinc, and protein, as well as biomarkers such as albumin, inflammatory markers, and phosphate, during the first two weeks of wound healing. These ratios can be implemented in clinical settings by hospitals through standardized guidelines to properly treat patients with BN and AN based on their specific physiological profiles. Personalized refeeding adjustments have the potential for shortening hospital stays; RCTs would also be beneficial in this area. In the same vein, future research should also specifically address age-related protocols for pediatric and adolescent groups, given that these populations are more susceptible to developing EDs and also due to the unique nutritional demands that growth and development have on nutritional necessities [29,30].

An emerging area not yet explored in ED surgical patients is immune nutrition. This practice focuses on supplementing specific nutrients such as arginine, glutamine, and omega3-fatty-acids to support angiogenesis and collagen synthesis during perioperative management [36,37]. In clinically malnourished surgical patients, immune-nutrition supplements have demonstrated a link to fewer postoperative complications including shorter hospital stays and a reduction in infection rates [38]. That said, no studies have yet tested immune-nutrition protocols in AN and BN patients undergoing surgery, despite this population’s known nutritional depletion and vulnerability to infection. This represents a clear, large gap in literature that should be investigated to determine if immune-nutrition supplementation can be safely used for this understudied population.

## Conclusions

Patients with EDs remain an understudied population in surgical settings and face unique risks due to the systemic pathophysiological cascade that chronic malnutrition causes. Collagen synthesis and skin integrity are compromised, leading to weakened immune function and exacerbating preventable postoperative events, including increased suture inflammation, PUs, SSIs, and fluid shifts that cause refeeding edema. Thus, tailored perioperative nutrition plans and ethical multidisciplinary protocols, such as preoperative SGA screening and postoperative NICE-guideline-based refeeding plans, are imperative to pair with electrolyte monitoring while replenishing protein and micronutrients (zinc and vitamin C) to optimize recovery times for patients affected with EDs. Per the NICE guidelines, initial refeeding should begin at 10 to 20 kcal/kg/day with gradual increases, aiming for weight gain of 0.43 to 0.86 kg/week, as guided by NICE guidelines. Due to its nature of providing complementary recommendations, the 2025 AuSPEN consensus statement should be considered when managing perioperative risks for ED patients. Future studies should include RCTs, as they are necessary to develop ED-specific perioperative protocols to both standardize care and reduce morbidity rates in this vulnerable patient population. If these challenges can be addressed, long-term physical and psychological recovery in ED patients can be optimized, reducing long hospital stays and life-threatening emergencies.
